# Hypothalamic KNDy neuron expression in streptozotocin-induced diabetic female rats

**DOI:** 10.1530/JOE-21-0169

**Published:** 2022-01-27

**Authors:** Hiroyuki Enomoto, Kinuyo Iwata, Keisuke Matsumoto, Mai Otsuka, Akio Morita, Hitoshi Ozawa

**Affiliations:** 1 Department of Anatomy and Neurobiology, Graduate School of Medicine, Nippon Medical School, Bunkyo-ku, Tokyo, Japan; 2 Department of Neurosurgery, Graduate School of Medicine, Nippon Medical School, Bunkyo-ku, Tokyo, Japan

**Keywords:** Kiss1, Tac3, Pdyn, diabetes mellitus, metastin, arcuate nucleus, anteroventral periventricular nucleus, luteinizing hormone, reproduction

## Abstract

Kisspeptin neurons, i.e. KNDy neurons, in the arcuate nucleus (ARC) coexpress neurokinin B and dynorphin and regulate gonadotropin-releasing hormone/luteinizing hormone (LH) pulses. Because it remains unclear whether these neurons are associated with reproductive dysfunction in diabetic females, we examined the expression of KNDy neurons detected by histochemistry in streptozotocin (STZ)-induced diabetic female rats 8 weeks after STZ injection. We also evaluated relevant metabolic parameters – glucose, 3-hydroxybutyrate, and non-esterified fatty acids – as indicators of diabetes progression. Severe diabetes with hyperglycemia and severe ketosis suppressed the mRNA expression of KNDy neurons, resulting in low plasma LH levels and persistent diestrus. In moderate diabetes with hyperglycemia and moderate ketosis, kisspeptin-immunoreactive cells and plasma LH levels were decreased, while the mRNA expression of KNDy neurons remained unchanged. Mild diabetes with hyperglycemia and slight ketosis did not affect KNDy neurons and plasma LH levels. The number of KNDy cells was strongly and negatively correlated with plasma 3-hydroxybutyrate levels. The vaginal smear analysis showed unclear proestrus in diabetic rats 3–5 days after STZ injection, and the mRNA expression of kisspeptin in the ARC was decreased 2 weeks after STZ injection in severely diabetic rats. Kisspeptin neurons in the anteroventral periventricular nucleus (AVPV), which induce an LH surge, were unaffected at 2 and 8 weeks after STZ injection regardless of the diabetes severity. These results suggest that diabetes mellitus progression in females may negatively affect ARC kisspeptin neurons but not AVPV kisspeptin neurons, implicating a potential role of ARC kisspeptin neurons in menstrual disorder and infertility.

## Introduction

Encoded by the *Kiss1* gene, kisspeptin and its receptor, G protein-coupled receptor 54, stimulate the release of gonadotropin-releasing hormone (GnRH)/luteinizing hormone (LH) in mammals and are crucial in ovulation regulation and follicle development (Roa *et al.* 2011). In rodents, kisspeptin neurons are mainly located in the anteroventral periventricular nucleus (AVPV) and the arcuate nucleus (ARC). Kisspeptin neurons in the AVPV are targets of estrogen-positive feedback and are thought to be involved in inducing the preovulatory GnRH/LH surge. Conversely, kisspeptin neurons in the ARC mediate the negative feedback effects of sex steroids on GnRH/LH secretion (Smith *et al.* 2005, Adachi *et al.* 2007, Iwata *et al.* 2017, Kanaya *et al.* 2017). In many mammals, ARC kisspeptin neurons coexpress neurokinin B (NKB) and dynorphin (Dyn), which are encoded by *Tac3* and *Pdyn* genes, respectively (Wakabayashi *et al.* 2010, Murakawa *et al.* 2016). These cells, termed KNDy neurons, are thought to be involved in generating pulsatile GnRH/LH secretion (Lehman *et al.* 2010).

Diabetes can cause erectile dysfunction in men and infertility and menstrual irregularities in women (Gandhi *et al.* 2017). Several studies examined hypothalamic kisspeptin neurons in diabetic rats. In male rats, *Kiss1* mRNA in the hypothalamus and the number of kisspeptin-immunoreactive (ir) cells in the ARC of streptozotocin (STZ)-induced diabetic rats remained unchanged 1–2 weeks after STZ injection (Castellano *et al.* 2009, Dudek *et al.* 2016). However, *Kiss1* mRNA in the hypothalamus decreased in male rats with long-term diabetes (4 weeks after STZ injection) (Castellano *et al.* 2006, 2009). In male rats with diabetes induced by both STZ and high-fat diet (HFD) with decreased plasma levels of endogenous testosterone, the number of kisspeptin-, NKB-, and Dyn-ir cells in the ARC was higher than in non-diabetic control rats (Dudek *et al.* 2017). In contrast, few studies have investigated diabetic female rats. The number of kisspeptin-, NKB-, and Dyn-ir cells in the ARC remained unchanged in female rats with diabetes induced by both STZ and HFD (Ziarniak *et al.* 2018). In another report, the number of Dyn-ir cells in the ARC was increased in diabetic female rats (Ziarniak *et al.* 2020). Additionally, *Kiss1* mRNA expression in the whole hypothalamus was reduced 4 weeks after STZ injection in female rats with long-term diabetes (Castellano *et al.* 2009). No studies have examined the number of *Kiss1*-, *Tac3*-, and *Pdyn* mRNA-expressing cells in the ARC and *Kiss1*-expressing cells in the AVPV in diabetic female animals. In women, the onset mechanism of diabetic-induced irregular menstruation is not fully elucidated, so we aimed to determine whether hypothalamic kisspeptin, NKB, and Dyn neurons were involved in female infertility due to diabetes. Therefore, we conducted the first study to determine the mRNA expression of these neurons in the ARC and *Kiss1* mRNA expression in the AVPV of STZ-induced diabetic female rats using *in situ* hybridization (ISH). Additionally, we measured the plasma levels of metabolic parameters as indicators of diabetes progression and investigated the relationships among the metabolic and reproductive parameters in these rats.

## Materials and methods

### Animals

The present study was approved by the Committee on Animal Research at Nippon Medical School. All experiments were performed according to the NIH Guide for the Care and Use of Laboratory Animals and approved by the Animal Care and Experimentation Committee at Nippon Medical School. Seven-week-old female Wistar-Imamichi rats (Institute for Animal Reproduction, Ibaraki, Japan) were housed in a controlled environment (14-h light:10-h darkness, 6:00 h lights on; 24°C ± 2°C; 50% humidity) with free access to food and water. Animals with at least two consecutive estrus cycles were used.

### Experimental design

#### Experiment 1

To produce mild-to-severe diabetes models, diabetes in 9-week-old rats was induced by a single i.v. or intraperitoneal (i.p.) injection of STZ (Merck), which destroys pancreatic β cells. STZ was used at 60 or 80 mg/kg body weight (BW) in 0.01 M citrate buffer (pH 4.5) to create the following groups: ST60-i.v., ST60-i.p., ST80-i.v., and ST80-i.p. Non-diabetic control animals received i.v. or i.p. citrate buffer only, forming the C-i.v. and C-i.p. groups, respectively. The estrous stage was identified by daily vaginal lavage for 15 or 16 days 5 weeks after STZ injection. After vaginal smear observation, all animals underwent ovariectomy (OVX) and were immediately implanted with a silicon tube (1.57-mm inner diameter, 3.18-mm outer diameter, 25 mm in length; Dow Corning, Midland, MI, USA) filled with 20 μg/mL 17β-estradiol (E2, Sigma–Aldrich) in sesame oil (Sigma–Aldrich) to equalize the effect of ovarian steroids. The E2 concentration used in the present study was previously shown to be necessary to produce a negative feedback effect on LH release (Cagampang *et al.* 1991). One week after the surgery (at 17 weeks of age), the jugular vein blood was collected under isoflurane anesthesia (2–3% in air) and the animals were perfused with 4% paraformaldehyde (PFA) for ISH (*Kiss1*, *Tac3*, and *Pdyn*) and immunohistochemistry (IHC; kisspeptin). The blood samples were utilized for LH, glucose, 3-hydroxybutyrate (3HB), and non-esterified fatty acids (NEFA) assays. All images were captured with a light microscope (BX51; Olympus) equipped with a charge-coupled device camera (DP73; Olympus) adapted to the microscope. The number of mRNA-expressing or ir cells in the captured images was counted using NIH ImageJ software v1.49b (https://imagej.nih.gov/ij/). The total number of cells in the brain sections was also determined.

#### Experiment 2

A silicon cannula (0.5-mm inner diameter; 1-mm outer diameter; Shin-Etsu Polymer, Tokyo, Japan) was inserted into the right atrium of 8- to 9-week-old rats with intact ovaries. On the next day, approximately 150 μL of blood was collected through the cannula, followed by the injection of STZ (60 or 80 mg/kg BW) or citrate buffer. Approximately, 150 μL of blood was collected once daily (9:00–10:00 h) after STZ injection through the cannula until the clogging of the cannula, which prevented blood collection. Daily blood collection and drug injection were performed on freely moving conscious rats. BW and food and water intakes were measured daily, and the estrous stage was identified by daily vaginal lavage for 14 days after STZ injection. After vaginal smear observation, blood from the jugular vein was collected under isoflurane anesthesia, followed by perfusion with 4% PFA for ISH and IHC at the diestrus stage.

### LH assay

The blood samples were immediately centrifuged, and the plasma was stored at −20°C. Plasma LH concentrations were measured using a rat-specific double-antibody RIA kit (National Hormone and Peptide Program, Baltimore, MD, USA). Values were expressed in terms of the National Institute of Diabetes and Digestive and Kidney Diseases rat LH RP-3. The minimum detectable level was 0.156 ng/mL in 50 μL plasma. The intra-assay coefficient of variation was 5.4% at 1.35 ng/mL. All samples (50 μL) were assayed in duplicate in a single assay.

### Energy substrate assays

Commercial kits were used to determine plasma levels of glucose (Glucose C2; FUJIFILM Wako Pure Chemical Corporation), 3HB (3-Hydroxybutyrate Assay Kit; Serotec, Sapporo, Japan), and NEFA (NEFA C; FUJIFILM Wako Pure Chemical Corporation). The plasma samples (glucose, 1.5 μL; NEFA, 5 μL; 3HB, 6.4 μL) were mixed with the reagents supplied with the kits. Glucose and NEFA were measured using a microplate absorbance reader (iMark; Bio-Rad). 3HB levels were measured using a multimode microplate reader (FilterMax F5; Molecular Devices, San Jose, CA, USA). All samples were assayed in duplicate in a single assay.

### Plasma E2 assay

A commercial estradiol ELISA kit (Cayman Chemical) was used to determine plasma E2 levels. Briefly, 100 μL of plasma and standard samples were extracted three times using diethyl ether and dissolved in 100 μL of assay buffer. The extracted samples were assayed in duplicate according to the kit instructions.

### Tissue preparation

After blood sample collection under isoflurane anesthesia, the rats were additionally anesthetized with a mixture of midazolam, butorphanol tartrate, and medetomidine hydrochloride. The rats were transcardially perfused with saline solution followed by 4% PFA in 0.1 M phosphate buffer (pH 7.4). The excised brains were immersed in the same fixative at 4°C overnight and then in 0.05 M phosphate buffer containing 20% sucrose at 4°C for 3 days for cryoprotection. Serial coronal sections (50-μm thick) were cut using a cryostat (CM3050 S; Leica) and divided into two series in the AVPV, from approximately −0.6 to 0.6 mm posterior to the bregma, and into four series in the ARC, from approximately 1.56 to 4.36 mm posterior to the bregma (Paxinos & Watson 2006). Each brain section series included approximately 14 and 16 sections in the AVPV and ARC, respectively.

### ISH for *Kiss1*, *Tac3*, and Pdyn

Free-floating ISH was performed to detect *Kiss1*, *Tac3*, and *Pdyn* mRNA, as previously described (Adachi *et al.* 2007, Kunimura *et al.* 2017). Digoxigenin (DIG)-labeled antisense complementary RNA probes were synthesized for *Kiss1* (position 39–527; GenBank accession no. XM_017598697) (Minabe *et al.* 2021), *Tac3* (position 180–483; GenBank accession no. NM019162) (Mostari *et al.* 2013, Minabe *et al.* 2021), and *Pdyn* (position 315–731; GenBank accession no. NM019374) (Mostari *et al.* 2013, Minabe *et al.* 2021) using a DIG RNA Labeling Kit (Roche Diagnostics). Briefly, the sections were hybridized overnight with each probe (1 μg/mL) at 60°C. The next day, after washing, the sections were treated with 20 μg/mL RNase A for 30 min at 37°C, washed, incubated with alkaline phosphatase-conjugated anti-DIG antibody (1:1000, Roche Diagnostics) for 1 h at 37°C, and stained with NBT/BCIP Stock Solution (1:50, Roche Diagnostics) for 1 h at room temperature.

### IHC for kisspeptin

Free-floating IHC was performed to detect kisspeptin, as previously described (Kunimura *et al.* 2017). Briefly, the sections were incubated overnight with mouse monoclonal anti-kisspeptin antibody (1:5000; Cat# 254, RRID: AB_2636957; kindly provided by Takeda Pharmaceutical Company, Osaka, Japan) (Kinoshita *et al.* 2005) and 5% normal rabbit serum in PBS with Tween 20 at 4°C. On the next day, the sections were treated with kit reagents (Histofine SAB-PO kit; Nichirei Biosciences, Tokyo, Japan) and stained with 3,3′-diaminobenzidine tetrahydrochloride (0.5 mg/mL, Sigma–Aldrich) in 0.01% H_2_O_2_. The same procedure was used for IHC controls without the specific primary antibody, and the absence of non-specific binding was confirmed (Murakawa *et al.* 2016).

### Statistical analysis

All data were expressed as mean ±s.e. of the mean. Comparisons among the i.v. or i.p. groups were assessed by one-way ANOVA followed by Tukey’s test. Daily changes in the plasma metabolic markers were determined by one-way ANOVA followed by Tukey’s test. Changes in BW and food and water intakes were determined by two-way ANOVA (group and week) followed by Bonferroni* post hoc* test. *P*-values of <0.05 were considered statistically significant.

A principal component analysis was performed using the prcomp command in R statistical software v3.5.3 (R Core Team 2019). The data comprised all results of the study, namely 39 samples (C-i.p., *n*  = 10; ST60-i.p., *n*  = 3; ST80-i.p., *n*  = 6; C-i.v., *n*  = 5; ST60-i.v., *n*  = 8; ST80-i.v., *n*  = 7) and 12 variables (BW; ovarian weight; percentage of diestrus during smear observation; plasma levels of LH, glucose, 3HB (ketone body), and NEFA; number of *Kiss1*-, *Tac3*-, and *Pdyn*-expressing cells; number of kisspeptin-ir cells in the ARC; and number of Kiss*1* cells in the AVPV). A dataset of 12 variables was obtained from a rat. The two variables (diestrus (%) and ovary weight) were obtained in intact rats, whereas the remaining variables were obtained after OVX+E2. Ovarian weight was measured at the time of OVX. Data on the percentage of diestrus were obtained during the 15- to 16-day period immediately before OVX. Data from two rats in ST60-i.p. were excluded due to the lack of *Tac3* and *Pdyn* results.

## Results

### Reproductive parameters of ovary-intact diabetic rats

**Figure 1:**
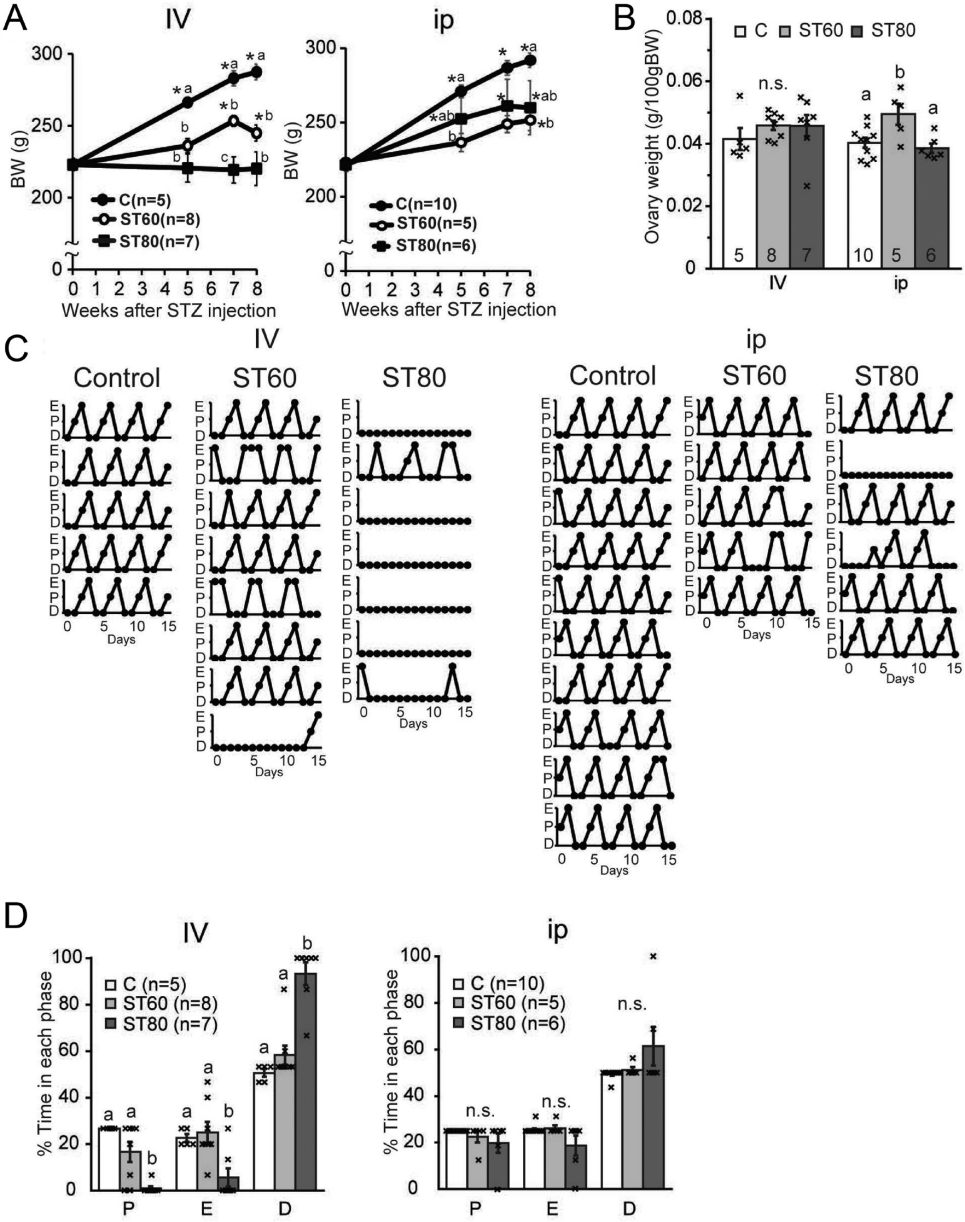
Reproductive parameters of ovary-intact diabetic rats. (A) Body weight (BW) after intravenous (i.v.; left) or intraperitoneal (i.p., right) administration of streptozotocin (STZ; 60 mg/kg BW, ST60; 80 mg/kg BW, ST80; non-diabetic control, C). Statistical differences were determined by two-way ANOVA (group and week), followed by Bonferroni correction. There was a significant interaction between the two factors (group and week) in i.v. and i.p. groups. Values with different letters and asterisks show significant BW differences at each week and vs BW just before STZ injection, respectively (*P* < 0.05). (B) Ovary weight (g/100 g BW) 7 weeks after STZ injection. (C) Cytological profiles of vaginal lavage of all animals. The estrous cycle phase was determined by the major cell population in the vaginal smear: proestrus (P), estrus (E), and diestrus (D). (D) Proportion of each phase in the estrous cycle. Statistical differences were determined by one-way ANOVA followed by Tukey’s test (B, D). Values with different letters are significantly different among each group (*P* < 0.05). Numbers in each column or in parentheses represent the number of rats examined. Values are expressed as mean ±s.e. of the mean. Marks in each bar graph show individual values. n.s., not significant.

In the i.v. group, the BW of ST80-i.v. and ST60-i.v. was significantly lower than C-i.v. after the STZ injection (Fig. 1A). In the i.p. group, the BW of ST60-i.p. was significantly different from C-i.p. (Fig. 1A). The ovarian weight (g/100 g BW) was not significantly different among the i.v. group rats. In the i.p. group, the ovarian weight in ST60-i.p. was higher than in C-i.p. and ST80-i.p. (Fig. 1B).

Regarding the i.v. group, almost all rats in ST80-i.v. showed persistent diestrus; five of seven rats exhibited diestrus on all days during the vaginal smear observation (Fig. 1C). The duration of the diestrus stage was significantly extended in ST80-i.v. compared with C-i.v. and ST60-i.v. (Fig. 1D). In ST60-i.v., three rats showed irregular estrous cycles (Fig. 1C). No significant differences were observed among the estrous cycles of the i.p. group rats (Fig. 1D). However, two of six rats in ST80-i.p. showed irregular estrous cycles (Fig. 1C).

### Expression of KNDy neurons in the ARC and *Kiss1* neurons in the AVPV in E2-treated ovariectomized rats

To determine whether diabetes-induced irregular estrous cycle was caused by hypothalamic dysfunction rather than ovarian dysfunction, E2-treated ovariectomized rats were used to equalize the effects of endogenous steroid hormones, given that the expression of kisspeptin, NKB, and Dyn in the hypothalamus is affected by sex steroid hormones.

**Figure 2:**
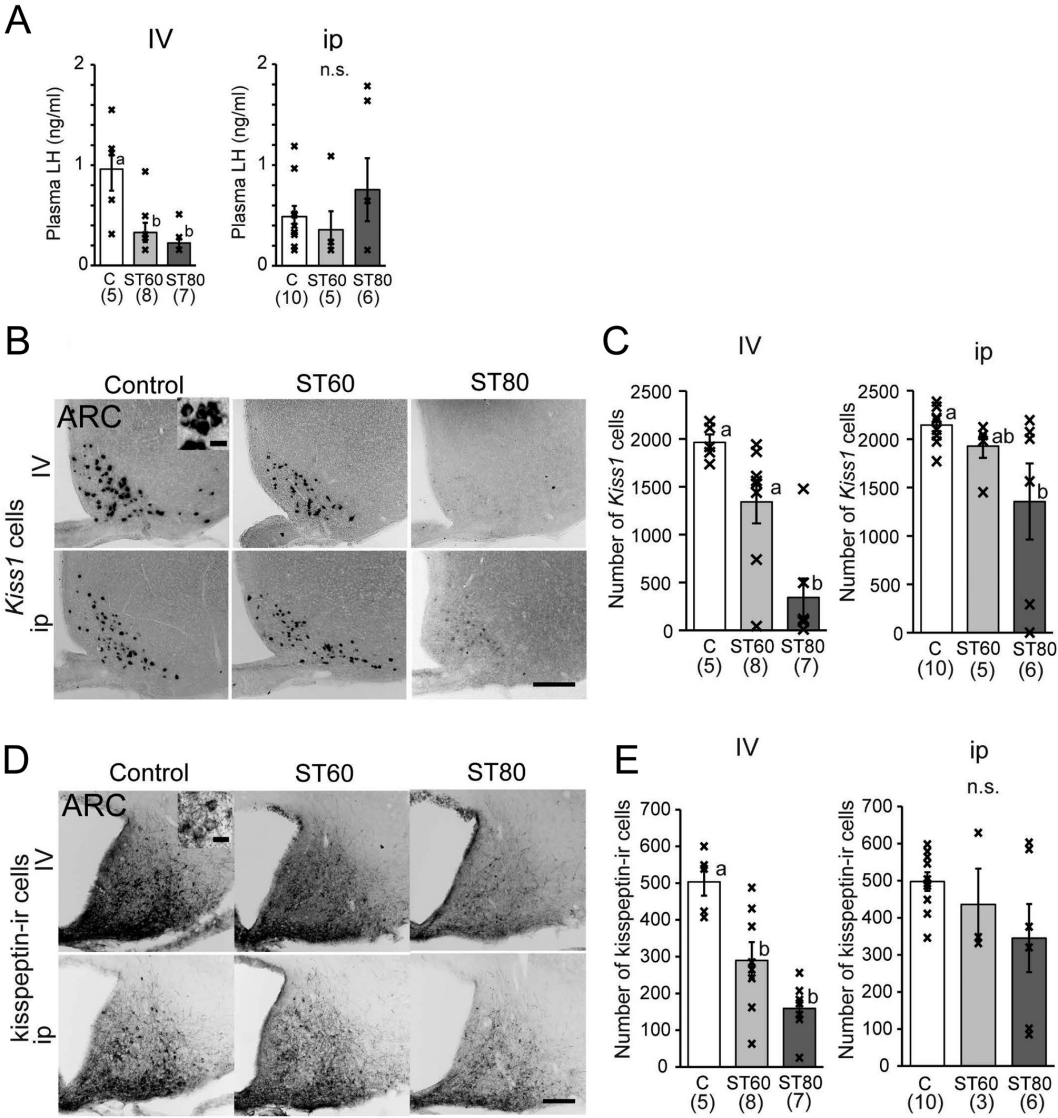
Expression of *Kiss1* by ISH and kisspeptin by IHC in the arcuate nucleus (ARC) of E2-treated ovariectomized rats. (A) Luteinizing hormone (LH) levels after OVX and E2 treatment. (B, C) Representative photos and the number of *Kiss1* mRNA-expressing cells in the ARC. (D, E) Representative images and the number of kisspeptin-immunoreactive (ir) cells in the ARC. Scale bar = 200 μm. Two insets show higher magnification of certain cells. Scale bar of insets = 20 μm. Numbers in parentheses under each column represent the number of rats examined. Values with different letters are significantly different among each group (*P* < 0.05, one-way ANOVA followed by Tukey’s test). Values are expressed as mean ±s.e. of the mean. Marks in each bar graph show individual values. n.s., not significant.

Plasma LH levels were significantly lower in ST60-i.v. and ST80-i.v. rats than in C-i.v. rats that underwent OVX treatment and received E2 (Fig. 2A). On the other hand, plasma LH levels were not significantly different among the i.p. group rats (Fig. 2A). However, the LH levels of two rats with irregular estrous cycles in ST80-i.p. were lower than the minimum detectable level.

**Figure 3:**
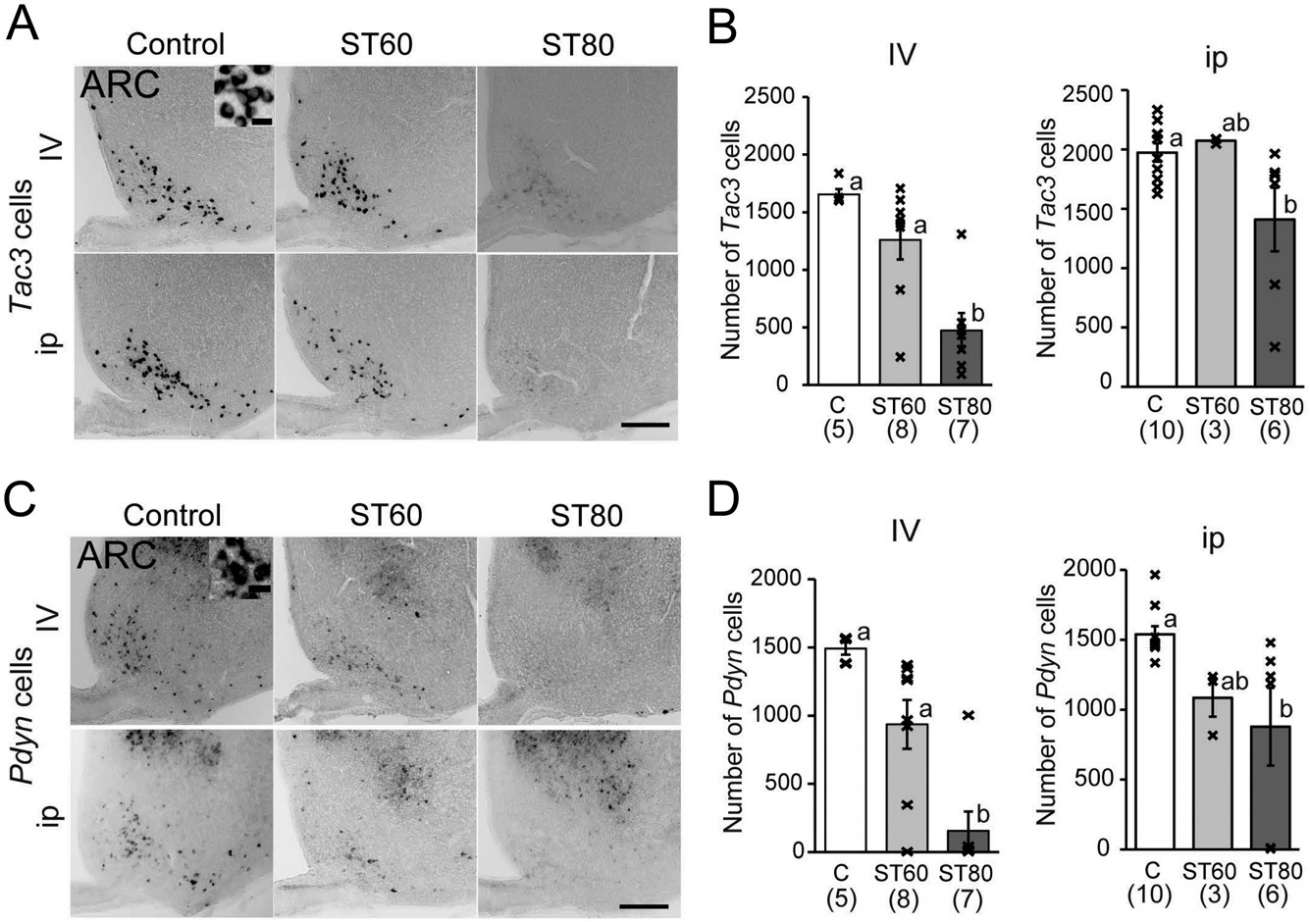
Expression of *Tac3* and *Pdyn* by ISH in the arcuate nucleus (ARC) in E2-treated ovariectomized rats. (A, B) Representative photos and the number of *Tac3* mRNA-expressing cells in the ARC. (C, D) Representative images and the number of *Pdyn* mRNA-expressing cells in the ARC. Scale bar = 200 μm. Two insets show higher magnification of certain cells. Scale bar of insets = 20 μm. Numbers in parenthesis under each column represent the number of rats examined. Values with different letters are significantly different among each group (*P* < 0.05, one-way ANOVA followed by Tukey’s test). Values are expressed as mean ±s.e. of the mean. Marks in each bar graph show individual values.

Regarding the ARC, the number of *Kiss1* mRNA-expressing cells in the ARC was significantly decreased in ST80-i.v. compared with C-i.v. and ST60-i.v. (Fig. 2B and  C). In the i.p. group, the number of Kiss*1* cells in ST80-i.p. was also significantly lower than in C-i.p. (Fig. 2B and  C). Kisspeptin-ir cells in the ARC were significantly decreased in ST80-i.v. and ST60-i.v. compared with C-i.v. (Fig. 2D and  E). However, there were no significant differences in kisspeptin-ir cells among the i.p. group rats (Fig. 2D and  E). The number of *Tac3* and *Pdyn* cells in the ARC was significantly decreased in ST80-i.v. relative to C-i.v. and ST60-i.v. (Fig. 3A, B, C and  D). No significant differences in *Tac3* and *Pdyn* cells were observed between ST60-i.v. and C-i.v., but one rat with persistent diestrus in ST60-i.v. showed profound decreases in *Kiss1*, *Tac3*, *Pdyn*, and kisspeptin-ir cells in the ARC. Another rat of ST60-i.v. also showed decreases in *Kiss1*, *Tac3*, and *Pdyn* cells, but it experienced regular estrous cycles. In the i.p. group, the number of *Tac3* and *Pdyn* cells in ST80-i.p. was significantly lower than in C-i.p. (Fig. 3A, B, C and  D). Two rats with irregular estrous cycles in ST80-i.p. exhibited profound decreases in *Kiss1*, *Tac3*, *Pdyn*, and kisspeptin-ir cells in the ARC.

**Figure 4:**
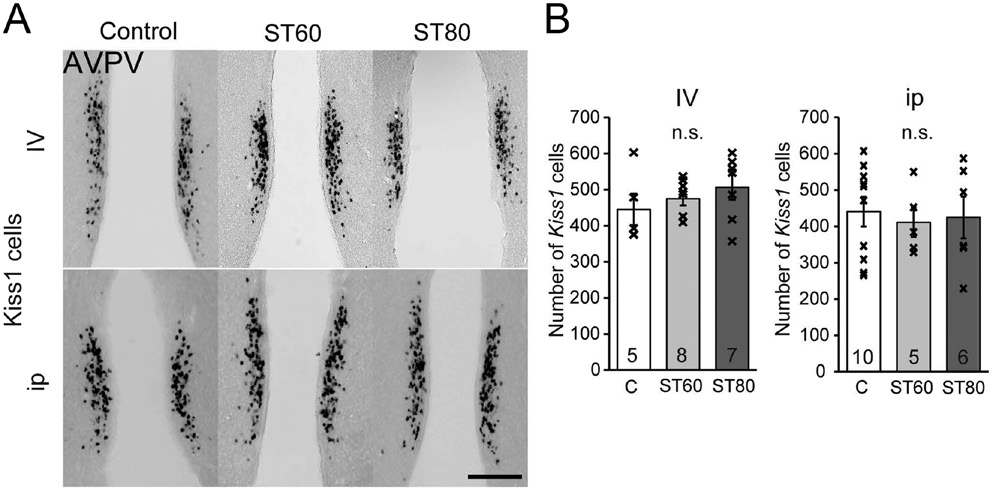
Expression of *Kiss1* by ISH in the anteroventral periventricular nucleus (AVPV) of E2-treated ovariectomized rats. (A, B) Representative photos and the number of *Kiss1* mRNA-expressing cells in the AVPV. Scale bar = 200 μm. Numbers in each column represent the number of rats examined. Comparisons among each group were assessed by one-way ANOVA followed by Tukey’s test (*P* < 0.05). Values are expressed as mean ±s.e. of the mean. Marks in each bar graph show individual values. n.s., not significant.

In contrast to the ARC, the number of Kiss*1* cells in the AVPV, which induce an LH surge, did not differ significantly among each group (Fig. 4).

### Plasma metabolic parameters in E2-treated ovariectomized rats

**Figure 5:**
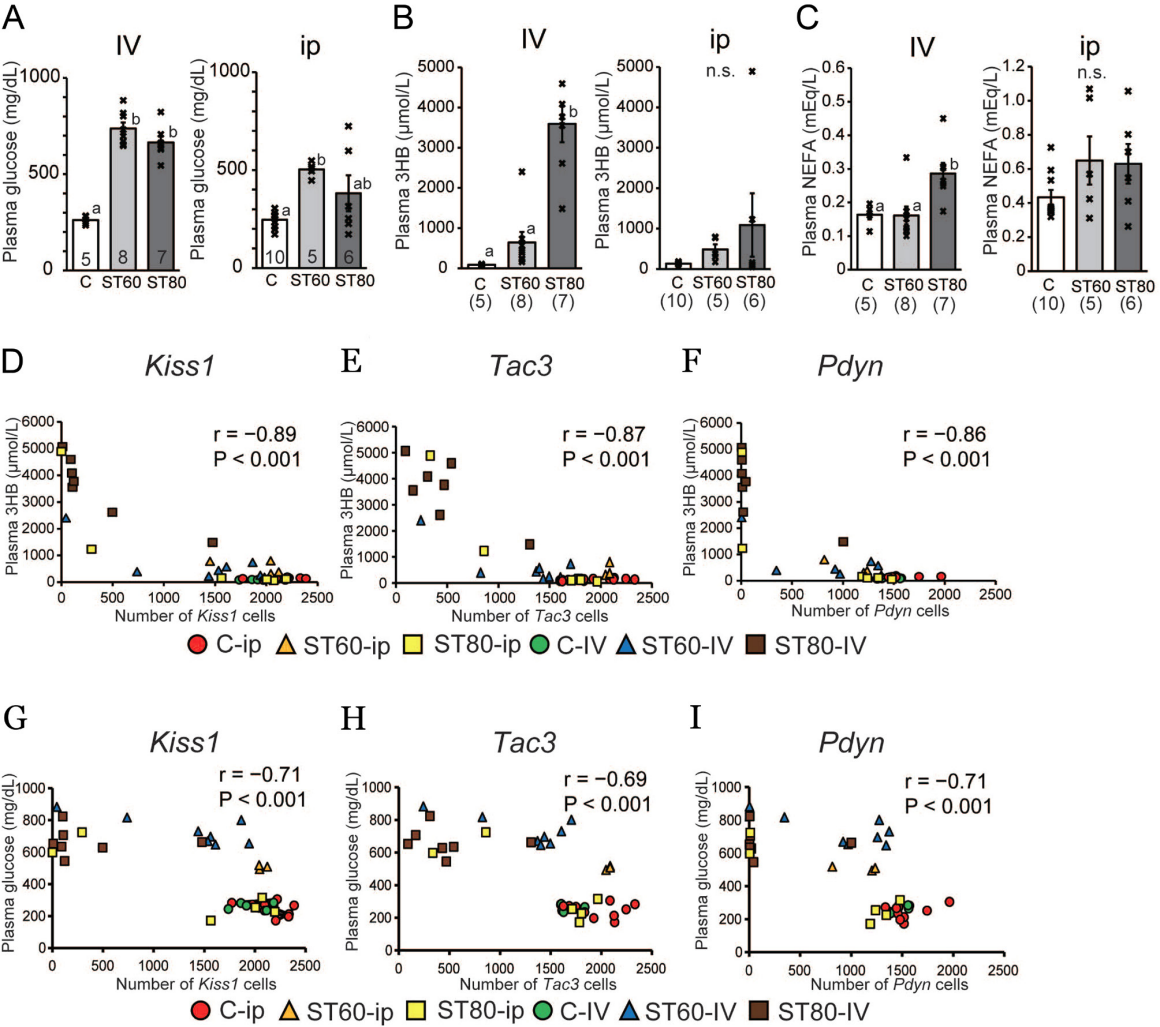
Metabolic parameters in the plasma of E2-treated ovariectomized rats and the relationship of KNDy neurons expressing kisspeptin, NKB, and Dyn in the ARC with plasma 3-hydroxybutyrate, glucose, and non-esterified fatty acid levels. (A, B and C) Levels of energy substrates in plasma samples (A: glucose; B: 3-hydroxybutyrate (3HB); C: non-esterified fatty acids (NEFA)). Blood samples were collected under anesthesia immediately before paraformaldehyde perfusion and immediately centrifuged for plasma collection. Statistical differences were determined by one-way ANOVA followed by Tukey’s test (A, B, and C). Values with different letters are significantly different among each group (*P* < 0.05). Numbers in each column or parentheses represent the number of rats examined. Values are expressed as mean ±s.e. of the mean. Marks in each bar graph show individual values. n.s., not significant. (D, E, and F) Plasma 3HB levels were used as an indicator of diabetes progression. Note the correlation between plasma 3HB levels and the number of *Kiss1-*(D), *Tac3-*(E), and *Pdyn*-expressing (F) cells in non-diabetic and diabetic rats. *r*, Pearson’s product–moment correlation coefficient. (G, H, and I) Correlation between plasma glucose levels and the number of *Kiss1-*(G), *Tac3-*(H), and *Pdyn*-expressing (I) cells in non-diabetic and diabetic rats.

Plasma glucose, 3HB, and NEFA levels were measured to evaluate diabetes progression. Plasma glucose levels were significantly higher in ST60-i.v. and ST80-i.v. than in C-i.v. (Fig. 5A). In the i.p. group, glucose levels were higher in ST60-i.p. than in C-i.p. (Fig. 5A). Plasma 3HB levels were significantly higher in ST80-i.v. than in ST60-i.v. and C-i.v. (Fig. 5B). Although a significant difference in 3HB was not observed between ST60-i.v. and C-i.v., mean 3HB levels were higher in ST60-i.v. than in C-i.v. (647 ± 259.9 vs 87 ± 5.8 μmol/L). There was no difference in plasma 3HB levels among the i.p. group rats (Fig. 5B); however, plasma 3HB levels tended to be higher in ST60-i.p. than in C-i.p. (487 ± 125.9 vs 136 ± 10.2 μmol/L). Plasma NEFA levels were significantly increased in ST80-i.v. compared with C-i.v. and ST60-i.v. and did not significantly differ among the i.p. group rats (Fig. 5C). Interestingly, the two rats in ST80-i.p. that showed irregular estrous cycles and low LH levels exhibited hyperglycemia (>500 mg/dL), severe ketosis (>1000 μmol/L), and high NEFA concentrations (the highest two values in ST80-i.p.). One rat with persistent diestrus in ST60-i.v. also exhibited low LH levels below the minimum detectable level, hyperglycemia (>800 mg/dL), and severe ketosis (>2000 μmol/L).

We also investigated the correlation between KNDy neurons in the ARC and plasma metabolic parameters as indicators of diabetes progression. The number of KNDy cells was strongly and negatively correlated with 3HB and glucose (Fig. 5D, E, F, G, H and  I). Importantly, the negative correlation of KNDy neurons with 3HB was stronger than that with glucose (correlation coefficients: 3HB, from −0.86 to −0.89; glucose, from −0.69 to −0.71).

### The relationship among metabolic/reproductive parameters

**Figure 6:**
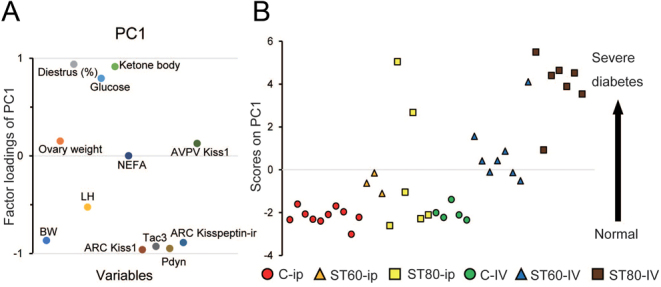
Principal component analysis. The data consist of 39 samples and 12 variables (body weight (BW); ovary weight; the percentage of diestrus throughout the smear observation; luteinizing hormone (LH); glucose; a ketone body (3-hydroxybutyrate); non-esterified fatty acids (NEFA); arcuate nucleus (ARC) *Kiss1*, *Tac3*, *Pdyn*, and kisspeptin-immunoreactive (ir) cells; and anteroventral periventricular nucleus (AVPV) *Kiss1*). (A) Scatter plot of factor loadings of the first component (PC1). Factor loadings range from 1 to −1. Factor loadings close to −1 or 1 indicate that the variables strongly influence PC1. Factor loadings close to zero indicate that the variables weakly influence the PC1. The PC1 accounts for 57.4% of the total variation. (B) Scatter plot of the 39 score values (C-i.p., *n*  = 10; ST60-i.p., *n*  = 3; ST80-i.p., *n*  = 6; C-i.v., *n*  = 5; ST60-i.v., *n*  = 8; ST80-i.v., *n*  = 7) in PC1. As diabetes severity progresses, scores on PC1 become higher and are plotted toward the top of the plot.

We performed a principal component analysis to reveal relationships among variables and among groups. The first component (PC1) accounted for 57.4% of the total variation. Diestrus (%), glucose, and ketone bodies were generally correlated in approximately 60% of the total variation (Fig. 6A). These three variables were also strongly correlated with PC1. BW; ARC *Kiss1*, *Tac3*, *Pdyn*, and kisspeptin-ir cells; and LH were correlated in approximately 60% of the total variation. These six variables were strongly negatively correlated with the PC1. Additionally, the three variables and the six variables, respectively, were perfectly, oppositely correlated (Fig. 6A). Thus, the score values on PC1 increased with increases in the percentage of diestrus, plasma levels of glucose, and 3HB and also increased with decreases in the number of *Kiss1*, *Tac3*, *Pdyn*, and kisspeptin-ir cells in the ARC; BW; and plasma LH. Thus, the scores on PC1 were considered to reflect the development of diabetes. In contrast, ovary weight, NEFA, and AVPV Kiss*1* cells, whose loadings were close to zero on PC1, hardly influenced the PC1 (Fig. 6A).

Figure 6B shows the scatter plot of the 39 score values on PC1. The score values were plotted toward the top of the plot with the progression of diabetes. The scores of almost all rats in ST80-i.v., one rat in ST60-i.v., and two rats in ST80-i.p. were very high, suggesting that diabetes could likely develop in these animals. The scores of most rats in ST60-i.v. and one rat in ST80-i.v. were lower than those of the high-score rats but higher than the control rats (Fig. 6B). Thus, these rats could show moderate diabetes. The scores of all rats in ST60-i.p., one rat in ST80-i.p., and a few rats in ST60-i.v. were slightly higher than the control rats, suggesting that these animals might have developed a mild disease. Surprisingly, the scores of three rats in ST80-i.p. were almost equal to the control rats, suggesting that a single i.p. injection of STZ might not have affected these animals. In the i.v. group, diabetes appeared to have developed in a dose-dependent manner according to the amount of STZ that was administered. However, the i.p. group showed no relationship between the STZ dosage and diabetes progression.

### Changes in metabolic/reproductive parameters 14 days after STZ injection

**Figure 7:**
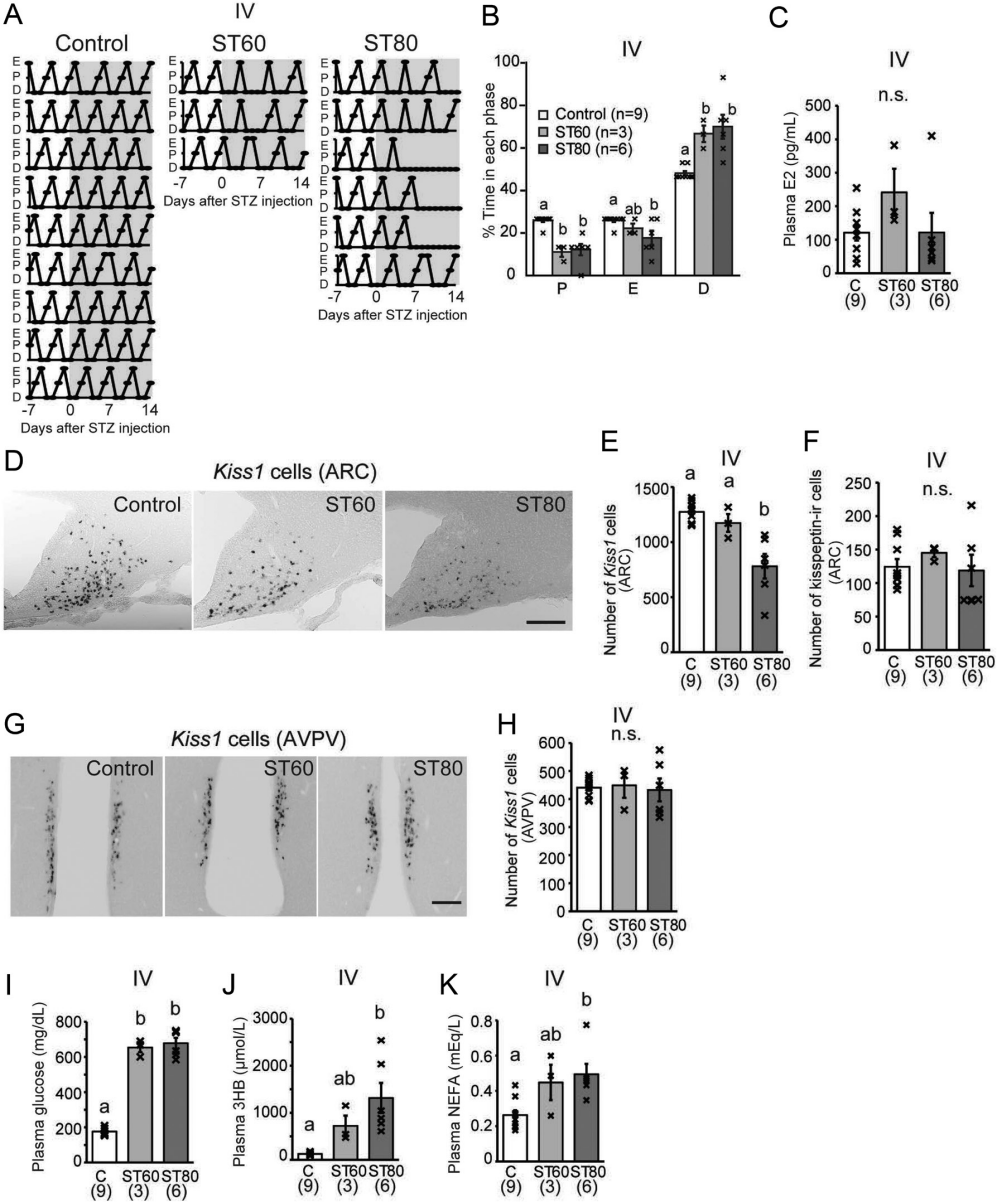
Reproductive parameters of ovary-intact rats 2 weeks after i.v. streptozotocin (STZ) injection. (A) Cytological profiles of vaginal lavage of all animals. STZ was injected on day 0 (C: non-diabetic control; ST60: STZ at 60 mg/kg BW; ST80: STZ at 80 mg/kg BW). The estrous cycle phase was determined based on the major cell population in the vaginal smear: proestrus (P), estrus (E), and diestrus (D). Diestrus was considered when the major cell population in the vaginal smear was nucleated cells and leucocytes such as unclear proestrus. (B) Proportion of each phase in the estrous cycle. (C) Plasma concentrations of estradiol (E2). (D and E) Representative images and the number of *Kiss1* mRNA-expressing cells in the arcuate nucleus (ARC). (F) The number of kisspeptin-immunoreactive (ir) cells in the ARC. (G and H) Representative images and the number of *Kiss1* mRNA-expressing cells in the anteroventral periventricular nucleus (AVPV). Scale bar = 200 μm. (I, J, and K) Energy substrates in plasma samples; (I) glucose, (J) 3-hydroxybutyrate (3HB), and (K) non-esterified fatty acids (NEFA). Numbers in parentheses under each column represent the number of rats examined. Values with different letters are significantly different among each group (*P* < 0.05, one-way ANOVA followed by Tukey’s test), (B, C, E, F, H, I, J, and K). Values are expressed as mean ± s.e. of the mean. Marks in each bar graph show individual values. n.s., not significant. Brains (D, E, F, G, and H) and plasma (C, I, J and K) were collected under anesthesia 2 weeks after STZ injection at diestrus stage.

The vaginal smears, which were checked daily after i.v. STZ injection to determine the development of irregular estrous cyclicity in STZ-induced diabetic rats, revealed unclear proestrus – i.e. the major cell population in the vaginal smear was nucleated cells and leucocytes – in ST60-i.v. and ST80-i.v. rats 3–5 days after STZ injection (Fig. 7A). The duration of diestrus was significantly longer in ST80-i.v. and ST60-i.v. compared with C-i.v. (Fig. 7B). Further, three out of six rats in ST80-i.v. exhibited persistent diestrus within 14 days after STZ injection. There were no significant differences in plasma E2 concentrations among the three groups (Fig. 7C).

*Kiss1* expression in ovary-intact animals was evaluated 2 weeks after STZ injection. The number of *Kiss1* mRNA-expressing cells in the ARC was significantly decreased in ST80-i.v. compared with C-i.v. and ST60-i.v. (Fig. 7D and  E). However, there was no significant difference in kisspeptin-ir cells in the ARC among the three groups (Fig. 7F). In the AVPV, there were no significant differences in the number of *Kiss1* mRNA-expressing cells among the three groups (Fig. 7G and  H). Plasma glucose levels 2 weeks after STZ injection (the day of perfusion) were significantly higher in ST60-i.v. and ST80-i.v. than in C-i.v. (Fig. 7I). Plasma 3HB and NEFA levels were significantly higher in ST80 than in C-i.v. (Fig. 7J and  K).

**Figure 8:**
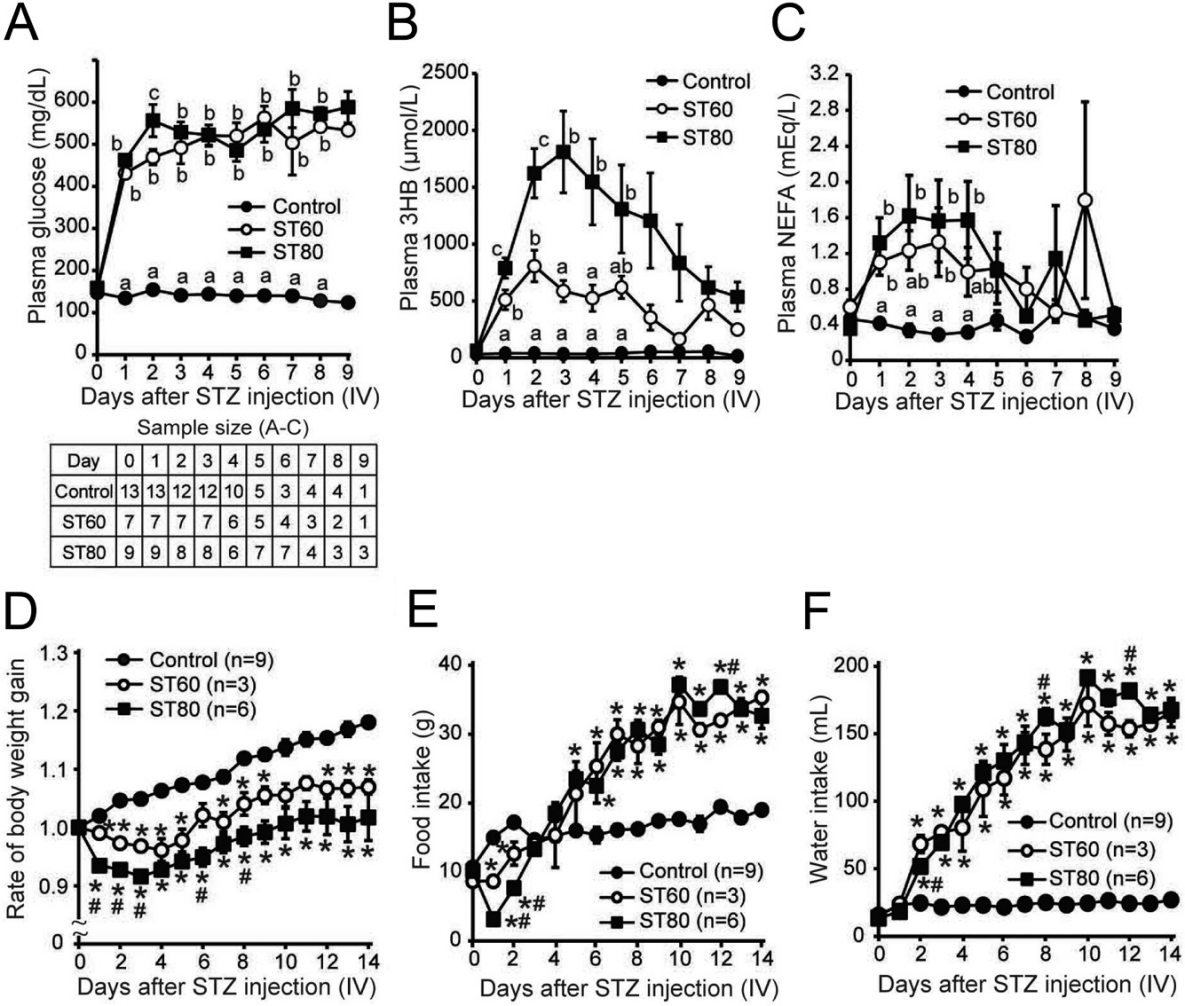
Daily changes in metabolic parameters after i.v. streptozotocin (STZ) injection. (A–C): Levels of energy substrates in plasma (A: glucose; B: 3-hydroxybutyrate (3HB); C: non-esterified fatty acids (NEFA)). Daily blood samples were collected from the jugular vein catheter of freely moving conscious rats. Statistical differences were determined by one-way ANOVA followed by Tukey’s test (A, B, and C). Values with different letters are significantly different among each group (*P* < 0.05). Values are expressed as mean ±s.e. of the mean. The accompanying table shows the sample size for data presented in A–C. (D) The rate of body weight gain. (E) Food intake. (F) Water intake. Statistical differences were determined by two-way ANOVA (group and day), followed by Bonferroni correction (D, E, and F). There was a significant interaction between the two factors (group and days), and the main effect of each factor (group or days) was statistically significant (D, E, and F). *P < 0.05, vs control. ^#^*P* < 0.05, vs ST60.

The analysis of daily changes in the plasma levels of glucose, 3HB, and NEFA after STZ injection revealed that they were significantly higher in ST60-i.v. and ST80-i.v. than in C-i.v. on the day after STZ injection, i.e. day 1 (Fig. 8A, B and  C). The rate of BW gain was lower in ST80-i.v. than in C-i.v. on day 1 and thereafter (Fig. 8D). Food intake was significantly increased on days 6 and 5 in ST60-i.v. and ST80-i.v., respectively, compared with C-i.v. (Fig. 8E). Water intake on day 2 was significantly higher in ST60-i.v. and ST80-i.v. than in C-i.v. (Fig. 8F).

## Discussion

In this study, rats of ST80-i.v. showed severe diabetes with hyperglycemia and severe ketosis. Plasma NEFA levels in these animals were significantly higher than in the control group, indicating increased lipolysis and fatty acid oxidation. Additionally, these animals exhibited persistent diestrus, low levels of plasma LH, and the suppression of *Kiss1*, *Tac3*, and *Pdyn* mRNA expression in the ARC. A few animals in ST60-i.v. and ST80-i.p. with severe diabetes also exhibited suppression of these genes. These results suggest that severe diabetes induces the suppression of *Kiss1*, *Tac3*, and *Pdyn* expression in the ARC, which could lead to low plasma LH levels and persistent diestrus. A study showed that kisspeptin injection induced LH release in STZ-induced diabetic female rats, which exhibited decreased serum LH levels and decreased *Kiss1* expression in the whole hypothalamus (Castellano *et al.* 2009). In male rats with STZ-induced diabetes, which exhibited decreased serum LH and testosterone levels and reduced prostate weight, repeated kisspeptin administration induced persistent increases in circulating LH and testosterone levels and rescued the decrease in prostate and testis weights (Castellano *et al.* 2006). Female rats with >90%* Kiss1* knockout in the ARC exhibited suppressed pulsatile LH release and the rescue of >20% KNDy neurons recovered pulsatile LH release and follicular development in female rates with global *Kiss1* knockout (Nagae *et al.* 2021). Therefore, kisspeptin neurons in the ARC are considered to be associated with low LH levels in diabetic rats.

ST60-i.v. rats showed hyperglycemia and moderate ketosis compared with the controls, but the number of *Kiss1*-, *Tac3*-, and *Pdyn*-expressing cells in the ARC did not differ from the controls. In contrast, kisspeptin-ir cells in the ARC were decreased in ST60-i.v. relative to the controls, suggesting that translation was suppressed in ARC kisspeptin neurons rather than the suppression of mRNA expression, which might have led to low plasma LH levels. However, most of the ST60-i.v. rats showed normal estrous cycles. In this study, plasma samples for LH assay were collected at one timepoint, not as a series of samples over a period of time, so pulsatile LH secretion may not be completely inhibited in ST60-i.v. rats.

With the exception of two rats in ST80-i.p. with severe diabetes, most rats in ST80-i.p. and ST60-i.p exhibited hyperglycemia and mild ketosis relative to the control group, while several rats showed the same levels of glucose and 3HB as the control group. The expression of *Kiss1*, *Tac3*, and *Pdyn* cells in the ARC was significantly decreased in ST80-i.p. relative to the control group; these results appear to be due to the inclusion of the results of the two rats with severe diabetes. In ST60-i.p., the expression of *Kiss1*, *Tac3*, and *Pdyn* cells in the ARC was unaffected compared with the control group. It has been previously reported that diabetes induction in female rats by both STZ and an HFD did not affect the number of kisspeptin-, NKB-, and Dyn-ir cells in the ARC (Ziarniak *et al.* 2018). Thus, mild diabetes may not affect the hypothalamic–pituitary–gonadal (HPG) axis. In another study, the number of Dyn-ir cells in the ARC was increased in diabetic female rats induced by both STZ and an HFD (Ziarniak *et al.* 2020), suggesting that Dyn release may be inhibited by diabetes, resulting in Dyn accumulation in the cell bodies, which can be detected by IHC. These results suggest that the transcription and translation of *Kiss1*, *Tac3*, and *Pdyn* genes in the ARC could be suppressed in proportion to the severity of diabetes, leading to female infertility. However, evidence for a causal relationship between KNDy neurons and female diabetic infertility requires further elucidation.

This is the first study to demonstrate *Kiss1* expression in the AVPV of diabetic female rats. In contrast to the ARC, the number of Kiss*1* cells in the AVPV did not differ among the members in both the i.v. and i.p. groups, so the expression of *Kiss1* mRNA in the AVPV may be unaffected by diabetes. In agreement with this study, the number of AVPV Kiss*1* cells of other pathological model rats was the same as that of control groups under OVX and E2 treatment, while ARC *Kiss1* expression was decreased in the models (Iwata *et al.* 2017, Nakao *et al.* 2018). Thus, AVPV kisspeptin neurons could respond to estrogen even if ARC kisspeptin neurons were suppressed by metabolic stress and sex steroid hormones. Further studies are necessary to clarify whether an LH surge is induced by estrogen treatment in severely diabetic female rats.

We also found changes in the levels of metabolic/reproductive parameters during the 2-week period after i.v. STZ injection. The rapid changes in metabolic parameters after STZ injection were followed by polydipsia, with a subsequent increase in food intake. Surprisingly, the estrus cycle exhibited unclear proestrus within a few days after STZ injection, and *Kiss1* expression in the ARC was suppressed in ovary-intact diabetic rats 2 weeks after STZ injection. Previous studies reported that serum testosterone levels were decreased in diabetic male rats 1 week after STZ injection (Castellano *et al.* 2009). Therefore, the suppression of *Kiss1*, *Tac3*, and *Pdyn* expression in severely diabetic rats 8 weeks after STZ injection observed in the present study might be rapidly induced by STZ administration and might not be a gradual induction in proportion to the severity of diabetes.

To our knowledge, this is the first study to document the relationship between plasma ketone body levels and the hypothalamic neurons regulating reproductive function. In clinical studies, women with type 1 diabetes (T1DM) exhibited an increase in the overall prevalence of menstrual disturbances compared with non-diabetic women (Kjaer *et al.* 1992, Yeshaya *et al.* 1995), and women diagnosed with T1DM before menarche had a higher probability of delayed menarche (Yeshaya *et al.* 1995). Additionally, women with T1DM and secondary amenorrhea had fewer LH pulses than non-diabetic women but responded to exogenous GnRH (South *et al.* 1993). However, to the best of our knowledge, no study to date has examined the relationship between ketosis/ketoacidosis/hyperketonemia and the hypothalamic neurons regulating reproductive function. In the present study, the ARC KNDy neurons were suppressed at 2 and 8 weeks after STZ treatment in severely diabetic rats with elevated levels of both 3HB and NEFA. Moreover, plasma 3HB levels exhibited a stronger negative correlation with KNDy neurons compared with glucose, although plasma glucose concentrations are often used as a major criterion for diabetes in animal models. Hence, plasma ketone body concentration might be a useful indicator for hypothalamic disruption in women with diabetes.

In conclusion, this study showed that KNDy neurons in the ARC were suppressed in proportion to diabetes severity. In contrast, the mRNA expression of AVPV kisspeptin neurons, which induce the preovulatory GnRH/LH surge, was unaffected by diabetes regardless of the disease progression. Thus, diabetes in females could negatively affect KNDy neurons in the ARC-regulating pulsatile LH secretion in proportion to diabetes progression, which might lead to menstrual disorder and infertility. Additionally, diabetes-induced changes in the HPG axis may vary depending on the severity of diabetes, suggesting that the severity of diabetes should be considered in the investigation of neurological problems.

## Declaration of interest

The authors declare that there is no conflict of interest that could be perceived as prejudicing the impartiality of the research reported.

## Funding

This work was supported by a Grant-in-Aid (grant number 18K06860) to H O from the Japan Society for the Promotion of Science http://dx.doi.org/10.13039/501100001691, Nippon Medical School Grant-in-Aid for Medical Research.

## Author contribution statement

H E performed experiments and wrote the manuscript. K I conceived the study, analyzed the data, and wrote and edited the manuscript. K M and M O performed experiments. A M and H O reviewed and edited the manuscript.

## References

[bib1] Adachi S Yamada S Takatsu Y Matsui H Kinoshita M Takase K Sugiura H Ohtaki T Matsumoto H Uenoyama Y 2007 Involvement of anteroventral periventricular metastin/kisspeptin neurons in estrogen positive feedback action on luteinizing hormone release in female rats. Journal of Reproduction and Development 53 367–378. (10.1262/jrd.18146) 17213691

[bib2] Cagampang FR Maeda KI Tsukamura H Ohkura S Ota K 1991 Involvement of ovarian steroids and endogenous opioids in the fasting-induced suppression of pulsatile LH release in ovariectomized rats. Journal of Endocrinology 129 321–328. (10.1677/joe.0.1290321) 2066689

[bib3] Castellano JM Navarro VM Fernández-Fernández R Roa J Vigo E Pineda R Dieguez C Aguilar E Pinilla L Tena-Sempere M 2006 Expression of hypothalamic KiSS-1 system and rescue of defective gonadotropic responses by kisspeptin in streptozotocin-induced diabetic male rats. Diabetes 55 2602–2610. (10.2337/db05-1584) 16936210

[bib4] Castellano JM Navarro VM Roa J Pineda R Sánchez-Garrido MA García-Galiano D Vigo E Dieguez C Aguilar E Pinilla L 2009 Alterations in hypothalamic KiSS-1 system in experimental diabetes: early changes and functional consequences. Endocrinology 150 784–794. (10.1210/en.2008-0849) 18845637

[bib5] Dudek M Kołodziejski PA Pruszyńska-Oszmałek E Sassek M Ziarniak K Nowak KW Sliwowska JH 2016 Effects of high-fat diet-induced obesity and diabetes on Kiss1 and GPR54 expression in the hypothalamic-pituitary-gonadal (HPG) axis and peripheral organs (fat, pancreas and liver) in male rats. Neuropeptides 56 41–49. (10.1016/j.npep.2016.01.005) 26853724

[bib6] Dudek M Kołodziejski PA Pruszyńska-Oszmałek E Ziarniak K Sliwowska JH 2017 Effects of orchidectomy and testosterone replacement on numbers of kisspeptin-, neurokinin B-, and dynorphin A-immunoreactive neurones in the arcuate nucleus of the hypothalamus in obese and diabetic rats. Journal of Neuroendocrinology 29. (10.1111/jne.12453) 28009489

[bib7] Gandhi J Dagur G Warren K Smith NL Sheynkin YR Zumbo A Khan SA 2017 The role of diabetes mellitus in sexual and reproductive health: an overview of pathogenesis, evaluation, and management. Current Diabetes Reviews 13 573–581. (10.2174/1573399813666161122124017) 27875946

[bib8] Iwata K Kunimura Y Matsumoto K Ozawa H 2017 Effect of androgen on Kiss1 expression and luteinizing hormone release in female rats. Journal of Endocrinology 233 281–292. (10.1530/JOE-16-0568) 28377404

[bib9] Kanaya M Iwata K Ozawa H 2017 Distinct dynorphin expression patterns with low- and high-dose estrogen treatment in the arcuate nucleus of female rats. Biology of Reproduction 97 709–718. (10.1093/biolre/iox131) 29069289

[bib10] Kinoshita M Tsukamura H Adachi S Matsui H Uenoyama Y Iwata K Yamada S Inoue K Ohtaki T Matsumoto H 2005 Involvement of central metastin in the regulation of preovulatory luteinizing hormone surge and estrous cyclicity in female rats. Endocrinology 146 4431–4436. (10.1210/en.2005-0195) 15976058

[bib11] Kjaer K Hagen C Sandø SH Eshøj O 1992 Epidemiology of menarche and menstrual disturbances in an unselected group of women with insulin-dependent diabetes mellitus compared to controls. Journal of Clinical Endocrinology and Metabolism 75 524–529. (10.1210/jcem.75.2.1639955) 1639955

[bib12] Kunimura Y Iwata K Ishigami A Ozawa H 2017 Age-related alterations in hypothalamic kisspeptin, neurokinin B, and dynorphin neurons and in pulsatile LH release in female and male rats. Neurobiology of Aging 50 30–38. (10.1016/j.neurobiolaging.2016.10.018) 27842268

[bib13] Lehman MN Merkley CM Coolen LM Goodman RL 2010 Anatomy of the kisspeptin neural network in mammals. Brain Research 1364 90–102. (10.1016/j.brainres.2010.09.020) 20858464 PMC2992597

[bib14] Minabe S Iwata K Tsuchida H Tsukamura H Ozawa H 2021 Effect of diet-induced obesity on Kiss1/Tac3/Pdyn gene expressions in the arcuate nucleus and luteinizing hormone secretion in sex hormone-primed male and female rats. Peptides 142 170546. (10.1016/j.peptides.2021.170546) 33794282

[bib15] Mostari P Ieda N Deura C Minabe S Yamada S Uenoyama Y Maeda K Tsukamura H 2013 Dynorphin-kappa opioid receptor signaling partly mediates estrogen negative feedback effect on LH pulses in female rats. Journal of Reproduction and Development 59 266–272. (10.1262/jrd.2012-193). PMC3934128 23391862

[bib16] Murakawa H Iwata K Takeshita T Ozawa H 2016 Immunoelectron microscopic observation of the subcellular localization of kisspeptin, neurokinin B and dynorphin A in KNDy neurons in the arcuate nucleus of the female rat. Neuroscience Letters 612 161–166. (10.1016/j.neulet.2015.12.008) 26679227

[bib17] Nagae M Uenoyama Y Okamoto S Tsuchida H Ikegami K Goto T Majarune S Nakamura S Sanbo M Hirabayashi M 2021 Direct evidence that KNDy neurons maintain gonadotropin pulses and folliculogenesis as the GnRH pulse generator. PNAS 118 e2009156118. (10.1073/pnas.2009156118) PMC7865162 33500349

[bib18] Nakao K Iwata K Takeshita T Ozawa H 2018 Expression of hypothalamic kisspeptin, neurokinin B, and dynorphin A neurons attenuates in female Zucker fatty rats. Neuroscience Letters 665 135–139. (10.1016/j.neulet.2017.12.002) 29203206

[bib19] Paxinos G Watson C 2006 The Rat Brain in Stereotaxic Coordinates, 6thed. Elsevier.(ISBN: 0-12-547612-4 )

[bib20] R Core Team 2019 R: a language and environment for statistical computing [Online]. (available at: https://www.R-project.org/)

[bib21] Roa J Navarro VM Tena-Sempere M 2011 Kisspeptins in reproductive biology: consensus knowledge and recent developments. Biology of Reproduction 85 650–660. (10.1095/biolreprod.111.091538) 21677307

[bib22] Smith JT Cunningham MJ Rissman EF Clifton DK Steiner RA 2005 Regulation of Kiss1 gene expression in the brain of the female mouse. Endocrinology 146 3686–3692. (10.1210/en.2005-0488) 15919741

[bib23] South SA Asplin CM Carlsen EC Booth RA Weltman JY Johnson ML Veldhuis JD Evans WS 1993 Alterations in luteinizing hormone secretory activity in women with insulin-dependent diabetes mellitus and secondary amenorrhea. Journal of Clinical Endocrinology and Metabolism 76 1048–1053. (10.1210/jcem.76.4.8473380) 8473380

[bib24] Wakabayashi Y Nakada T Murata K Ohkura S Mogi K Navarro VM Clifton DK Mori Y Tsukamura H Maeda K 2010 Neurokinin B and dynorphin A in kisspeptin neurons of the arcuate nucleus participate in generation of periodic oscillation of neural activity driving pulsatile gonadotropin-releasing hormone secretion in the goat. Journal of Neuroscience 30 3124–3132. (10.1523/JNEUROSCI.5848-09.2010) 20181609 PMC6633939

[bib25] Yeshaya A Orvieto R Dicker D Karp M Ben-Rafael Z 1995 Menstrual characteristics of women suffering from insulin-dependent diabetes mellitus. International Journal of Fertility and Menopausal Studies 40 269–273.(https://pubmed.ncbi.nlm.nih.gov/8556032/) 8556032

[bib26] Ziarniak K Kołodziejski PA Pruszyńska-Oszmałek E Kallό I Śliwowska JH 2018 High-fat diet and type 2 diabetes induced disruption of the oestrous cycle and alteration of hormonal profiles, but did not affect subpopulations of KNDy neurones in female rats. Journal of Neuroendocrinology 30 e12651. (10.1111/jne.12651) 30311288

[bib27] Ziarniak K Kołodziejski PA Pruszyńska-Oszmałek E Dudek M Kalló I Śliwowska JH 2020 Effects of ovariectomy and sex hormone replacement on numbers of kisspeptin-, neurokinin B- and dynorphin A-immunoreactive neurons in the arcuate nucleus of the hypothalamus in obese and diabetic rats. Neuroscience 451 184–196. (10.1016/j.neuroscience.2020.10.003) 33065232

